# Effect of Water Content in Semidry Grinding on the Quality of Glutinous Rice Flour

**DOI:** 10.3390/foods13203216

**Published:** 2024-10-10

**Authors:** Tao Huang, Dan Ouyang, Shangyuan Sang, Caiming Li, Xiaosan Wang, Xiao Wang, Jiali Xing, Xiaohu Luo

**Affiliations:** 1 Zhejiang-Malaysia Joint Research Laboratory for Agricultural Product Processing and Nutrition, College of Food Science and Engineering, Ningbo University, Ningbo 315832, China; 2211390052@nbu.edu.cn (T.H.); ouyangdan@nbu.edu.cn (D.O.); sangshangyuan@nbu.edu.cn (S.S.); 2 School of Food Science and Technology, Jiangnan University, Wuxi 214122, China; licaiming2009@126.com (C.L.); xiaosanw@jiangnan.edu.cn (X.W.); 3 Crop Breeding and Cultivation Research Institution, Research Center for Agricultural Products Preservation and Processing, Shanghai Academy of Agricultural Sciences, Shanghai 201400, China; wangxiao.0127@163.com; 4 Key Laboratory of Detection and Risk Prevention of Key Hazardous Materials in Food, China General Chamber of Commerce, Ningbo Key Laboratory of Detection, Control, and Early Warning of Key Hazardous Materials in Food, Ningbo Academy of Product and Food Quality Inspection (Ningbo Fibre Inspection Institute), Ningbo 315048, China

**Keywords:** glutinous rice flour, quality, semidry grinding, water content

## Abstract

The grinding process is one of the key factors affecting the quality of glutinous rice flour (GRF). As an emerging grinding method, semidry grinding aims to solve the problems of the high yield of wastewater in traditional wet grinding and the high content of damaged starch in dry grinding, in which the water content has a great influence on the quality of GRF. However, semidry grinding has not yet been formally put into production due to limitations such as the long time required to adjust the water content of rice grains. Therefore, this work was carried out to shorten the soaking time of glutinous rice (GR) by hot air pretreatment, and to conduct a systematic and in-depth study of the effect of water content on the quality of GRF, including water distribution, water hydration properties, thermal properties, rheological properties, and microstructure. The results showed that the GRF with higher water content had lower water solubility and higher enthalpy of pasting, which were due to the low content of damaged starch and the high degree of crystallization. The particle size of the GRF became smaller as the interaction between water and starch was enhanced and the GR was softened. In addition, the viscosity and elasticity of the GRF were also improved with an increase in water content. This work provides theoretical guidance for the improvement of semidry grinding to a certain extent.

## 1. Introduction

Glutinous rice (GR) is a type of rice containing a large amount of amylose with a soft texture, high viscosity, and excellent freeze–thaw stability, and is served as one of the staple foods in China, Japan, India, etc. [1]. In addition, GR is rich in nutrients, including proteins, essential fatty acids, amino acids, and vitamin B. Therefore, glutinous rice flour (GRF) is widely used as a raw material in the preparation of snacks and festive foods, such as sweet dumplings (Tang-yuan), rice cakes in China, sushi in Japan, and songpyeon in Korea [2]. Moreover, GRF is gluten-free and contains few allergens, making it useful and valuable in gluten-free foods in recent years [3].

Optimization of conditions during the grinding process is the key to obtaining GRF with excellent quality. Wet grinding, including cleaning, soaking, grinding, and drying, is the most traditional way of making GRF, and has been applied to long-term production practice. The benefit of GR soaking, namely, the hydration of cell walls and other polymers, is significant in wet grinding [4]. Consequently, water can be better immersed into the GR, resulting in minimizing mechanical damage during the grinding process [5]. However, the disadvantages of wet grinding are relatively evident, including a cumbersome technical process, long production cycle, and the generation of a large amount of wastewater, which is contrary to the increasing environmental protection requirements [6]. By contrast, dry grinding is a relatively simple way of making flour that involves removing impurities, crushing, and sieving. Considering that no water is involved during dry grinding, the GRF usually has a low water content, which facilitates long-term storage and transportation, and there is no need to worry about wastewater contamination [7]. Nevertheless, dry grinding generates huge amount of heat energy during grinding, which damages the nutritional structure of GR [8].

In order to solve the problems above, semidry grinding has been developed. Semidry grinding is used to address the large damage of the starch content in dry grinding and the large wastewater discharge in wet grinding [9]. This process mainly consists of the removal of impurities, water content adjustment, and grinding [10]. Recently, some studies combined dry and wet rice flour in a 1:1 ratio, but the improvement was not very remarkable [11]. In addition, some studies focused on the adjustment of water content, which was adjusted by soaking the rice grains in water for 24 h to reduce the damaged starch content to the level of wet grinding rice flour; however, prolonged soaking could result in food safety problems [12]. It has been shown that semidry grinding can effectively improve the quality characteristics of GRF, in which water content plays a crucial role [13]. However, few reports have systematically investigated water content on the quality of GRF with semidry grinding.

At present, the effect of the variation in water content during semidry grinding on the physiochemistry properties of rice flour is still unclear. Therefore, the objective of this work was to investigate the systematic effects of water transformation (tightly bound water, weakly bound water, and free water) on the physicochemical properties of GRF during semidry grinding by adjusting the moisture content of GR from low to high. We employed hot air pretreatment to shorten the soaking time required for GR. Then, the water content of GR in semidry grinding was further adjusted to investigate the effect on the quality of GRF. Specifically, the water distribution, water hydration, particle size, thermal properties, crystalline structure, rheology, and microstructure were utilized to evaluate the properties and quality of GRF. This study not only provides an important technological support for the transformation and upgrading of GR products, but also makes the commercial production of GRF more environmentally friendly and efficient.

## 2. Materials and Methods

### 2.1. Materials

GR was provided by Gang Yagou Food Co (Ningbo, China). The protein and fat content of GR was 7.2% and 0.7%, respectively. Water was deionized water. Potassium bromide (purity ≥ 99%) was purchased from Aladdin Bio-Chem Technology Co., Ltd. (Shanghai, China)

### 2.2. Determination of Soaking Temperature

A quantity of 100 g of GR was accurately weighed and pretreated with hot air at 45 °C for 60 min. Then, the GR was soaked with deionized water having different temperatures for 30 min (25, 50, 100 °C). The results of particle size and color properties are shown in Table S1, where the two properties were the worst at 100 °C. This was because at 100 °C, GR pasting occurred, which hindered the absorption of water molecules, causing the surface of the paste starch to agglomerate and making it hard to crush. Although the two properties were the best at 50 °C, soaking in warm water tends to breed mold [14]. Therefore, we ended up opting for water soaking at 25 °C.

### 2.3. Ash and Fibre Analysis

The ash content was determined with the method of Czaja, T. [15]. A quantity of 3 g of GRF was heated to 550 °C in a muffle furnace. This was burned until a light gray ash or constant weight was achieved. After the samples cooled, their weights were determined and the amount of ash was calculated (Table S2 in Supplementary Materials).

The fiber content was determined with the method of McCleary, B.V. [16]. A quantity of 2 g of GRF was placed in a crucible. Then, it experienced acid decoction and alkaline decoction, and was placed in a muffle furnace at 550 °C. The samples were burned until a light gray ash or constant weight was obtained. After cooling, the samples were weighed and the fiber content was calculated (Table S2 in the Supplementary Material).

The result showed that as the water content rose, so did the ash, while there was no significant difference in the fiber.

### 2.4. Semidry Grinding

A quantity of 100 g of GR was accurately weighed and pretreated with hot air at 45 °C for 60 min. Then, the rice was added with different contents of deionized water to soak for 30 min (0%, 16%, 20%, 24%, 28%, and 32%). The formula for the amount of water added was calculated as follows:

(1) $$\mathrm{Added}\;\mathrm{deionized}\;\mathrm{water}\;\left(g\right)=W(M_{1}-M_{0})\;/\;(1-M_{1})$$

where *W* represents the sample weight (g), *M*_0_ represents the original moisture (%), and *M*_1_ represents the target moisture (%).

After soaking the rice, the GR was ground into flour by a cyclone grinder (THFM110-11, TUOHE, Shanghai, China). After passing through a 100-mesh sieve (0.15 mm), the GRF was dried at 40 °C for 30 min to ensure its safe storage. The obtained GRF was loaded onto a sealed bag and stored at 4 °C for spare.

### 2.5. Low-Field Nuclear Magnetic Resonance (LF-NMR)

A 5.0 g sample was used for LF-NMR testing (Niumag Co., Ltd., Shanghai, China). The spin–spin relaxation time (T_2_) of the sample was measured by using the CPMG pulse sequence with the following parameters: main frequency SF = 21 MHz, 90-pulse RF pulse width P1 = 15 s, 180-pulse RF pulse width P2 = 25.04 s, receiver bandwidth SW = 200 kHz, and wave time TE = 0.2 ms. The LF-NMR signal was analyzed by software of LF-NMR instrument (version 4.0, NMI20-060H-1, Niumag Co., Ltd., Shanghai, China).

### 2.6. Water Hydration Properties

The water hydration properties of GRF were determined in accordance with the method of Shi et al. [17]. A quantity of 0.1 g of GRF was mixed with 20 mL of deionized water. Then, the mixture was heated and stirred using a magnetic stirrer (C-MAG HS 10 digital, IKA, Königswinter, Germany) for 30 min at 25 °C and 100 °C. The mixture was centrifuged at 8500× for 30 min. After centrifugation, the supernatant was dried at 105 °C until a constant weight was reached. The water absorption index (*WAI*), water solubility (*WS*), and swelling power (*SP*) were calculated as follows:

(2) WAI=wet sediment/dry sample weight

(3) $$WS\;\left(\%\right)=weight\;of\;supernatant/dry\;sample\times 100$$

(4) $$SP=wet\;sediment/[dry\;sample\;weight\times \left(100-WS\;\left(\%\right)/100\right)$$

### 2.7. Particle Size

The particle size of GRF was determined using a particle size analyzer pair (BT-9300S, Dandong Baxter Instrument Co., Ltd, Liaoning, China ) [18]. Deionized water was used as the optical model of light scattering. The sample (100 mg) was dispersed in 5 mL of water and stirred at 200 rpm for 10 min. Then, the dispersion was rapidly transferred to the cuvette of the particle size analyzer.

### 2.8. Differential Scanning Calorimetry (DSC) Analysis

The thermal properties of GRF were determined using a differential scanning calorimeter (DSC3500, NETZSCH, Bavaria State, Germany) in accordance with the method of Wang et al. [19]. A quantity of 5.0 mg of the GRF was accurately weighed in a DSC aluminum pan and mixed with 10 μL deionized water. After stabilizing at 4 °C for 24 h, the mixture was heated from 30 °C to 100 °C at the rate of 10 °C/min with an empty pan used as reference. The enthalpy change (∆H), onset temperature (T_o_), peak temperature (T_p_), and end temperature (T_e_) were recorded by using the software of the DSC instrument (version 8.0.3, NETZSCH, Bavaria State, Germany).

### 2.9. Fourier Transform-Infrared Spectroscopy (FT-IR)

FTIR spectra of GRF were obtained on a Fourier infrared spectrometer (Nicolet iS20, Thermo Fisher, Waltham, MA, USA) in accordance with the method of Zhai et al. [20]. GRF was processed by using the platen method. The spectral range was set in the range of 4000–400 cm^−1^ by accumulating 32 scans at a resolution of 4 cm^−1^. The infrared spectra were analyzed by Omnic software 8.2 (Thermo Fisher Scientific, Inc., Waltham, MA, USA), and the absorbance values at wavelengths of 1047, 1022, and 995 cm^−1^ were recorded.

### 2.10. X-ray Diffraction (XRD)

An Xpert PRO diffractometer (D8 Advance, Bruker Inc., Billerica, MA, USA) equipped with Cu-Kα radiation (λ = 0.1542 nm) was used to determine the crystalline structure of GRF in accordance with the methodology of Wang et al. [21]. Each sample was scanned at a speed of 10°/min and a step width of 0.033°, covering the range of 5° to 40° (2θ, the angle of diffraction). The crystallinity was calculated by Software Jade 6.0 (Materials Data, Inc., Billerica, MA, USA).

### 2.11. Rheological Properties

The viscoelasticity of the GRF was analyzed according to the method of Pan et al. [22] with slight modifications. The elastic modulus (G′) and viscous modulus (G″) of the GRF batters (20%, *w*/*w*) were recorded at an experimental temperature of 30 °C using a 40 mm flat plate with a measurement gap of 1 mm.

### 2.12. Microstructure

After freeze-drying, the GRF was carefully spread on double-sided sticky tape fixed to a copper stub. Then, the GRF was coated with gold–palladium. The micrographs were observed at 5.0 kV accelerating voltage and 1000× magnification. All the GRF was observed using a scanning electron microscopy (SEM, Hitachi S-570, Hitachi, Co., Ltd., Tokyo, Japan) at an acceleration voltage of 5.0 kV.

### 2.13. Statistical Analysis

All the experiments were independently conducted in triplicate, and data are expressed as mean ± standard deviation. All data were processes using the software Origin (version 9.0, Stat-Ease Inc., Minneapolis, MN, USA) and SPSS 19.0 (SPSS Inc., Chicago, IL, USA). Variations were considered to be significantly different at *p* < 0.05.

## 3. Results and Discussion

### 3.1. Analysis of the Water Status via LF-NMR

Water in GR could be categorized into three forms: tightly bound water T_21_ (0.01–1 ms), weakly bound water T_22_ (1–10 ms), and free water T_23_ (10–10,000 ms) [23]. GR samples with different water contents were investigated using LF-NMR, where T_2_ relaxation time reflected the internal chemical environment of the hydrogen proton, which was closely related to the binding force and the degree of freedom of the hydrogen proton. The larger the binding force or the smaller the degree of freedom, the shorter the T_2_ relaxation time.

**Figure 1:**
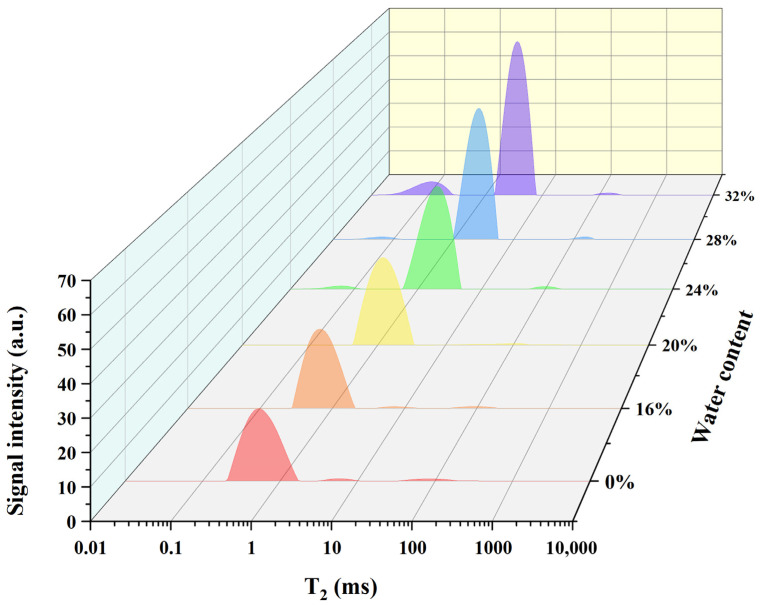
Water distribution in glutinous rice (GR) under different water contents.

As shown in Figure 1, the T_2_ peaks shifted to the right and the signal amplitude increased with the increase in water content, indicating that water gradually penetrated into the GR, so that the water content and water freedom of the GR increased. Although the peak area of T_21_ decreased, The T_21_ peak appeared at much smaller T_2_ values (0.01–0.1 ms), which suggested that the remaining tightly bound water became more closely integrated as the water content increased [24]. As a consequence, with the increase in water content, the water molecules in the GR system could be effectively bound, which in turn inhibited the starch coagulation.

In addition, the total peak area of T_23_ decreased, as shown in Figure 1, which was attributed to the transformation of free water into weakly bound water. There might be two main reasons for this transformation. On the one hand, some proteins in the periphery of GR and the hydrophilic groups present on its surface had a strong affinity for free water, which led to the combination of free water and proteins during rice soaking. On the other hand, with the increase in water content, the content of damaged starch decreased and the internal structure changed from an amorphous state to a more stable crystalline state, where water molecules were immobilized by incorporating into the recrystallized structure. This restricted the mobility and slowed down the migration of water molecules to a certain extent [25].

On the whole, the water inside the GR after soaking primarily existed in the form of weakly bound water, and the overall freedom of water decreased, allowing the GR to maintain a high water-holding capacity.

### 3.2. Water Hydration Properties of Glutinous Rice Flour

The characteristics of foods based on starch were significantly impacted by the hydration process of starch [26]. Hydration properties include WS, WAI, and SP. At 25 °C, the solubility of natural starch granules was low, and only a small amount of water could be captured due to the hydroxyl groups in the crystalline regions interacting through hydrogen bonding and little interaction with external water molecules [27]. As illustrated in Table 1, the water content had a significant effect on the WS of GRF at 25 °C, and the WS decreased with the increase in water content. This was because the GRF with higher water content had lower content of damaged starch, which was more prone to molecular degradation than intact natural starch, resulting in fewer soluble molecules, thus decreasing the WS of GRF [28]. Furthermore, this trend might also be due to the formation of more water-soluble short chains or leaching of more amorphous amylose from the damaged starch. The number of sorption sites of GRF subjected to severe mechanical shock decreased compared with that in the case of low mechanical shock [26]. Thus, GRF with small starch granules was subjected to less mechanical impact, had more adsorption points, which led a stronger adsorption capacity for water, and performed better in WS and WAI. The WAI of the soaked GRF showed a trend of first increasing and then decreasing. The water in the GR facilitated the interaction of non-starch components, which could increase the WAI. However, with the water content increasing, the damaged starch content decreased, resulting in a decrease in WAI. The same tendency was observed in the SP of soaked GRF with different water contents. The SP increased because the interaction between water and starch molecules was enhanced. However, the amylose could inhibit the granular swelling; with the water content increasing, amylose content increased, resulting in the enhancing of the rigid particle structure of GRF, which decreased the SP.

By contrast, at a pasting temperature of 100 °C, the WAI, WS, and SP values of soaked GRF were significantly decreased compared with those of dry GRF. This result might be attributed to the fact that the starch particles expand to the maximum at 100 °C, and the decrease in the damaged starch content would cause less soluble components to precipitate, causing the reduction in WS and SP. In addition, the low content of damaged starch means that the starch particles had a small relative surface area, and less binding with water, causing the reduction in WAI. The values of WAI, WS, and SP at 100 °C were higher than those at 25 °C, which indicated that the hydrogen bonds between the hydroxyl groups in double-helix starch molecules were damaged during starch pasting, allowing the hydroxyl groups to form new hydrogen bonds with water molecules, which led to the swelling of the starch granules [29].

### 3.3. Particle Size Properties of Glutinous Rice Flour

The average particle size of the GRF varied under different water contents. As displayed in Table 2, the particle size of GRF decreased with the increase in water content, where the average particle size of GRF with 32% water content was the smallest (11.45 μm), while the average particle size of GRF with 0% water content was the largest (16.88 μm). This might be explained by the fact that the rise in water content more thoroughly softened the soaked GR. Hence, it enabled more homogeneous grinding on the one hand, and on the other hand reduced the mechanical energy during grinding, so damaged starch content was reduced, which was also consistent with the data shown in Table 1. The decrease in damaged starch content resulted in a significant decrease in the swelling of starch, which is consistent with the findings of Jovin Hasjim et al. [29]. As described by Desam, G.P. et al. [30], swelling of starch occurred mainly at temperatures higher than the gelatinization temperature; the higher the temperature, the greater the swelling, thus resulting in a larger granule size. However, as the water content increased, the thermal energy generated by the grinding process decreased, so starch swelling was inhibited and the particle size showed a tendency to decrease. To the best of our knowledge, damaged starch was more easily hygroscopic and swollen compared to intact starch particles [31]. Accordingly, the swelling power of GRF decreased with the reduction in damaged starch content, which led to the decrease in the particle size of GRF.

### 3.4. Thermal Properties

Further, the thermal properties of GRF were evaluated. In DSC experiments, ΔH indicated the enthalpy required to melt starch granules and therefore reflected the degree of molecular disorder [32]. The results of DSC are exhibited in Table 2. ΔH of the soaked GRF increased significantly compared with the dry GRF, from 3.118 to 3.945 J/g, which illustrated that the content of damaged starch in the soaked GRF was reduced, indicating a higher degree of order. This was because the GRF with higher damaged starch content had more severe destruction in the crystalline structure and the double-helix structure [33]. Additionally, the results in Table 2 revealed that with the water content increasing, the initial temperature of gelatinization (T_o_) decreased significantly and the termination temperature (T_e_) increased generally compared with dry GRF, causing a wider temperature range for pasting. This was because of the low homogeneity of the distribution of damaged starch, which led to difficulties in pasting [34].

### 3.5. FTIR Analysis

**Figure 2:**
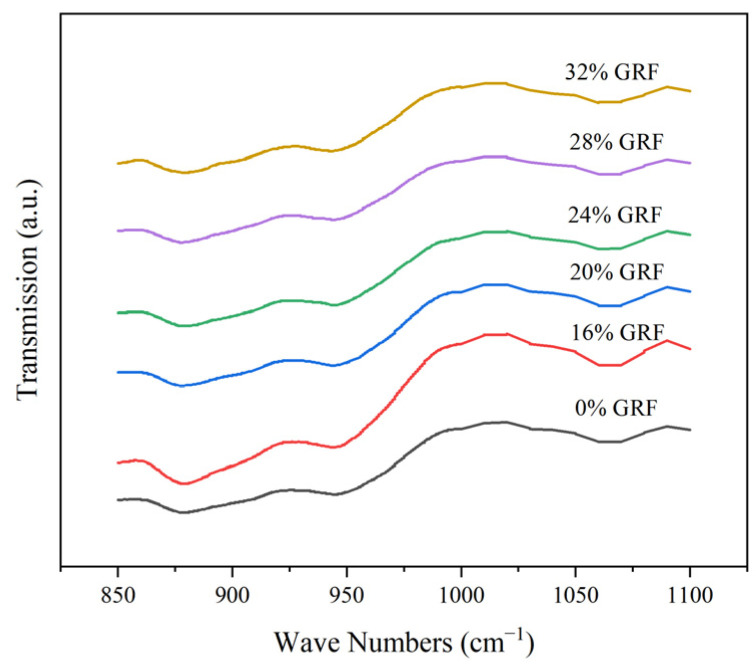
FTIR spectra of GRF with different water content.

FTIR spectroscopy was used to determine whether intermolecular hydrogen bonding was present in the sample. The result is shown in Figure 2. All samples had a broad absorption peak in the band between 3300 and 3700 cm^−1^, which was a typical stretching vibration absorption peak of the polysaccharide hydroxyl group. As the water content increased, the peak shifted to the lower wave numbers and the peak type gradually widened, indicating that the number of hydrogen bonds increased and the hydrogen bond effect was obviously enhanced [35].

The changes in the crystallinity in starch retrogradation were characterized by FTIR spectroscopy. R_1047/1022_ represents the degree of ordering of the starch sample, and the peak at 995 cm^−1^ was quite sensitive to water molecules in the samples [36]. As shown in Table 3, R_1047/1022_ of the soaked GRF increased compared with that of the dry GRF. This difference indicates that the increase in water content could promote the rearrangement of starch molecules and the recombination of damaged starch to form an ordered structure. In addition, 995 cm^−1^ increased from 0.123 to 0.276 with the increase in the water content, which indicated that GR with a high water content soaked more thoroughly, allowing the starch to fully integrate with the water.

### 3.6. Crystalline Structure

**Figure 3:**
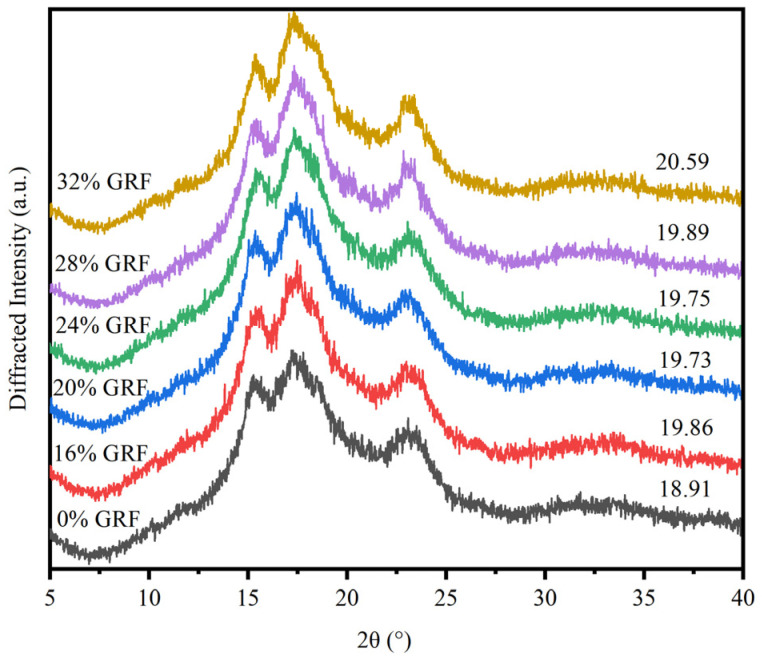
X-ray diffraction patterns of GRF with different water contents.

Figure 3 illustrates the XRD of GRF with different water contents. The samples showed an A-type crystal structure with distinct diffraction peaks at around 15°, 18°, and 23°. The polymorphic type did not change as the amount of water content increased, but the degree of crystallinity did. The relative degree of crystallinity increased from 18.91 to 20.59 as the water content increased, indicating an increase in relative crystallinity. As described by Asmeda, R. et al. [37], the GRF obtained by dry grinding demonstrated a greater impact of mechanical damage due to the high grain hardness of rice kernels, making it harder to pulverize during the grinding process, and hence causing greater damage to the starch granules. The increase in crystallinity indicates an increase in the orderliness of the crystal structure and an increase in the energy required to melt crystals during heating, which was consistent with the results of FTIR spectroscopy and the thermal properties. During the grinding process, the GRF with higher water content was subjected to less mechanical stress and water protected the GR, leading to a decrease in the content of damaged starch and resulting in a more complete crystal structure. Moreover, fewer hydrogen bonds between the side chains of amylopectin clusters were broken, thereby causing the increase in relative crystallinity.

### 3.7. Analysis of Rheological Properties

**Figure 4:**
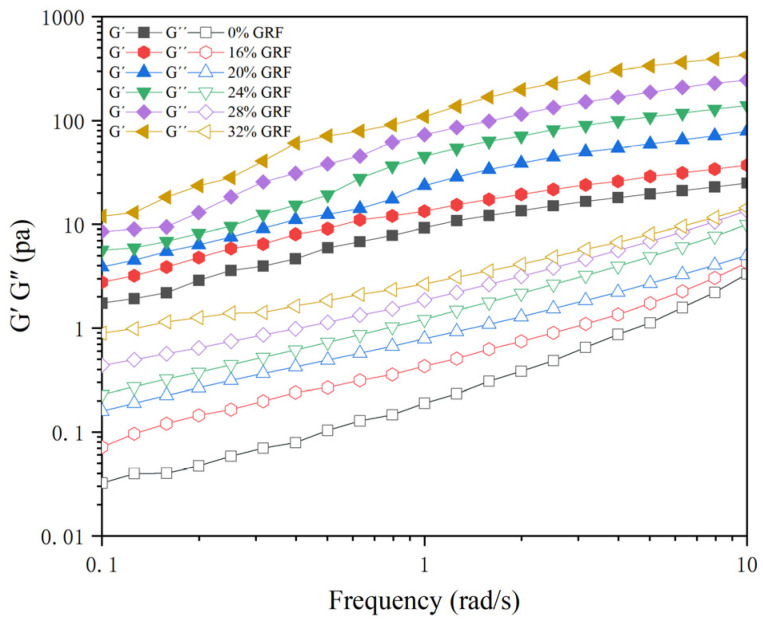
Variation in G′ and G″ values for GRF with different water contents.

**Figure 5:**
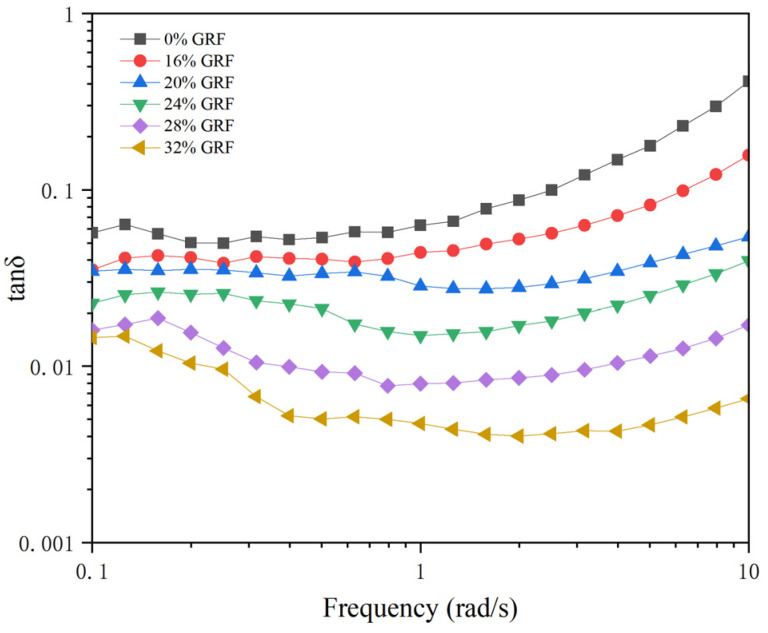
Variation in tanδ values for GRF with different water contents.

Frequency scanning is a common and important type rheological oscillation experiment used to characterize changes in the structure and viscoelasticity of starch gels [38]. As shown in Figure 4, the magnitude of the elastic modulus G′ and viscous modulus G″ of the GRF batters increased with the scanning frequency. The soaking treatment significantly changed the G′ and G″ values of the starch paste system, indicating that the change in starch structure during soaking ultimately led to a change in the structure of the starch gel. The G′ values of the soaked GRF batters were higher than those of the dried GRF batters, which was probably because the soaking with water content in the range of 16% to 32% facilitated the swelling of starch granules and the leaching of amylose. In addition, the formation of starch gel was mainly accomplished by the spatial rearrangement and aggregation of amylose. Therefore, the G′ values of the soaked GRF batters increased [39]. Similarly, the G′′ values of the soaked GRF batters were higher than those of the dry GRF batter and increased with increasing water content. Moreover, tanδ of all samples was less than unity (Figure 5), demonstrating the presence of typical weak gels. All tanδ values were low in the lower frequency range, indicating that the starch gel preferred to exhibit solid elastic properties. As the frequency increased, the tanδ of the samples rose, implying that more and more fluidic features were embodied in the system. However, it could be found that the increase in water content inhibited the rise in tanδ, suggesting that the effect of the soaking treatment on the elastic properties of starch gel was greater than the effect on the viscous properties of starch gel.

### 3.8. Scanning Electron Microscopy (SEM)

**Figure 6:**
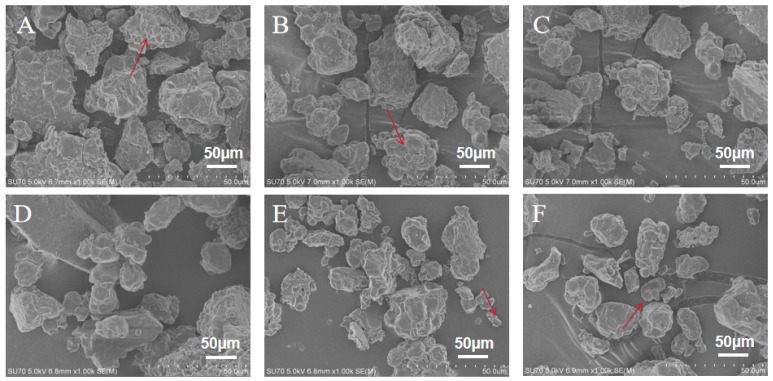
Morphology of GRF with different water contents: (**A**) 0% GRF; (**B**) 16% GRF; (**C**) 20% GRF; (**D**) 24% GRF; (**E**) 28% GRF; (**F**) 32% GRF. (The arrows showed that as the water content increased, the starch-protein aggregates became progressively smaller and scattered starch granules with contoured structures appeared.)

The morphology of GRF was observed by SEM, which revealed that the GRF presented an irregular polyhedron structure with varying sizes. As shown in Figure 6A, the GRF granules were large and rough without a clear starch profile, consisting of the aggregates of protein and starch. This was because when the water content is 0%, the grinding process generated a huge amount of mechanical energy, which caused damage to the structure of the GRF. A large amount of damaged starch appeared, so the surface was rough and inhomogeneous. With the increase in water content, the agglomerates of starch proteins gradually disintegrated and became smaller (Figure 6B–D). When the water content reached 28% and 32%, some scattered starch granules with contoured structures appeared (Figure 6E,F), which suggests that the semidry grinding process could reduce the production of damaged starch by the buffering effect on the mechanical damage, thus protecting the crystalline structure of starch granules.

## 4. Conclusions

In this work, the effect of water content on the quality of GRF during semidry grinding was investigated. The GR was soaked after hot air treatment, which could greatly reduce the soaking time and waste water contamination. On the other hand, the quality of GRF could be improved by adjusting the water content during rice soaking. From the data of hydration properties, it could be seen that the WS values of soaked GRF were significantly better than those of dried GRF and reached a maximum at 32% water content. In addition, similar results were found in ΔH of thermal properties and the relative crystallinity degree of XRD. This was because, with the increase in water content, the amount of damaged starch decreased, leading to a decrease in soluble molecules due to starch granules remaining relatively intact during the grinding process. Furthermore, the rise in water led to an increase in the interaction force between water and starch, which softened the GR and made the particle size of the GRF smaller. Moreover, based on the rheological properties, the G′ and G″ values of the GRF soaked with water were higher than those of the dried GRF, suggesting that the water content played an important role in maintaining the structure of the elastic and viscous properties of the GRF. This work can be expected to improve the semidry grinding process and promote the transformation and upgrading development of the grinding industry.

## Figures and Tables

**Table 1:** Hydration properties of GRF with different water contents at 25 °C and 100 °C.

Temperature (°C)	Water Content (%)	WAI	WS	SP
25	0	4.49 ± 0.03 c	5.67 ± 0.47 a	4.72 ± 0.30 c
	16	4.51 ± 0.36 c	4.90 ± 0.40 b	4.78 ± 0.39 bc
	20	4.89 ± 0.02 ab	4.03 ± 0.23 c	5.09 ± 0.03 ab
	24	5.11 ± 0.04 a	3.43 ± 0.23 d	5.30 ± 0.06 a
	28	4.77 ± 0.06 bc	2.77 ± 0.27 e	4.90 ± 0.05 bc
	32	4.58 ± 0.04 bc	2.20 ± 0.10 e	4.68 ± 0.03 c
100	0	15.30 ± 0.00 a	36.20 ± 1.70 a	23.95 ± 0.63 a
	16	13.10 ± 0.20 b	27.90 ± 0.90 b	18.19 ± 0.23 b
	20	12.78 ± 0.45 bc	13.77 ± 0.57 e	14.82 ± 0.46 d
	24	12.37 ± 0.17 c	25.90 ± 0.70 c	16.69 ± 0.32 c
	28	10.70 ± 0.60 d	12.87 ± 0.43 e	12.27 ± 0.72 e
	32	12.50 ± 0.58 bc	20.43 ± 0.93 d	15.71 ± 0.54 d

Mean values ± SD with different lowercase letters on shoulder tags in the same column indicate significant difference between them (*p* < 0.05).

**Table 2:** Thermal properties and particle size of GRF with different water content.

Water Content (%)	ΔH (J/g)	T_p_ (°C)	T_o_ (°C)	T_e_ (°C)	Particle Size (μm)
0	3.113 ± 0.017 d	66.95 ± 0.25 bc	60.16 ± 0.22 a	77.74 ± 0.71 d	16.88 ± 0.28 a
16	3.639 ± 0.042 bc	67.17 ± 0.26 ab	59.98 ± 1.09 a	78.61 ± 0.28 cd	16.08 ± 0.30 b
20	3.555 ± 0.002 c	67.65 ± 0.24 a	58.58 ± 0.45 b	80.54 ± 0.97 a	13.58 ± 0.25 c
24	3.594 ± 0.024 c	67.01 ± 0.48 bc	57.93 ± 1.13 b	80.05 ± 0.60 ab	13.42 ± 0.25 c
28	3.715 ± 0.078 b	66.73 ± 0.10 bc	56.81 ± 0.92 c	79.93 ± 0.86 ab	12.56 ± 0.26 d
32	3.945 ± 0.126 a	66.58 ± 0.29 c	56.85 ± 0.26 c	79.05 ± 0.63 bc	11.45 ± 0.25 e

Mean values ± SD with different lowercase letters on shoulder tags in the same column indicate a significant difference between them (*p* < 0.05).

**Table 3:** The 995 cm^−1^ infrared spectral values and the ratio of R_1047/1022_ of GRF with different water contents.

Water Content (%)	R_1047/1022_	995 cm^−1^
0	1.020 ± 0.003 d	0.123 ± 0.007 d
16	1.031 ± 0.002 c	0.232 ± 0.002 c
20	1.033 ± 0.003 c	0.260 ± 0.004 b
24	1.038 ± 0.008 b	0.265 ± 0.004 ab
28	1.041 ± 0.002 ab	0.270 ± 0.002 ab
32	1.045 ± 0.003 a	0.276 ± 0.007 a

Mean values ± SD with different lowercase letters in the same column indicate a significant difference between them (*p* < 0.05).

## Data Availability

The data presented in this study are available on request from the corresponding authors. The data are not publicly available due to privacy restrictions.

## Supplementary Materials

The following supporting information can be downloaded at: https://www.mdpi.com/article/10.3390/foods13203216/s1, Table S1: Color characteristics and particle size of GRF with different soaking temperature.; Table S2: Ash and Fibre content of GRF with different water content.
