# Supplementary material for: Effect of Water Content in Semidry Grinding on the Quality of Glutinous Rice Flour
Source: Foods. 2024 Oct 10;13(20):3216. doi: 10.3390/foods13203216 (PMC11507609; doi:10.3390/foods13203216)
Supplement: Supplementary file 1 [file foods-13-03216-s001.zip › foods-3198225-supplementary.pdf]

Supplementary material

Table S1. Color characteristics and particle size of GRF with different soaking temperature.

Soaking temperature(℃)	L*	a *	b *	particle size (μm)
25	91.86 ± 0.11b	0.22 ± 0.02a	2.91 ± 0.04a	11.45 ± 0.25b
50	92.38± 0.20a	0.24± 0.01a	2.68 ± 0.04b	10.35 ± 0.07c
100	91.22± 0.04c	0.20± 0.01a	3.38 ± 0.04c	13.60 ± 0.14a

Table S2. Ash and Fibre content of GRF with different water content.

water content (%)	Ash (%)	Fibre (%)
0	0.41 ± 0.02d	0.86 ± 0.02a
16	0.45 ± 0.01bc	0.86± 0.01a
20	0.47± 0.01bc	0.87 ± 0.04a
24	0.50 ± 0.02a	0.87 ± 0.03a
28	0.49 ± 0.01ab	0.88 ± 0.06a
32	0.51 ± 0.02a	0.89 ± 0.01a
